# A post-GWAS confirming effects of *PRKG1* gene on milk fatty acids in a Chinese Holstein dairy population

**DOI:** 10.1186/s12863-019-0755-7

**Published:** 2019-07-03

**Authors:** Lijun Shi, Xiaoqing Lv, Lin Liu, Yuze Yang, Zhu Ma, Bo Han, Dongxiao Sun

**Affiliations:** 1 Department of Animal Genetics, Breeding and Reproduction, College of Animal Science and Technology, Key Laboratory of Animal Genetics, Breeding and Reproduction of Ministry of Agriculture and Rural Affairs, National Engineering Laboratory for Animal Breeding, China Agricultural University, No. 2 Yuanmingyuan West Road, Haidian District, Beijing, 100193 China; 2 Beijing Dairy Cattle Center, Beijing, 100192 China; 3 Beijing Municipal Bureau of Agriculture, Beijing, 100101 China

**Keywords:** Dairy cattle, Effects of *PRKG1* gene, Fatty acid traits, Transcriptional activity

## Abstract

**Background:**

We previously conducted a genome-wide association study (GWAS) strategy for milk fatty acids in Chinese Holstein, and identified 83 genome-wide significant single nucleotide polymorphisms (SNPs) and 314 suggestive significant SNPs. Among them, two SNPs, BTB-01077939 and BTA-11275-no-rs associated with C10:0, C12:0, and C14 index (*P* = 0.000014 ~ 0.000024), were within and close to (0.85 Mb) protein kinase, cGMP-dependent, type І (*PRKG1*) gene on BTA26, respectively. *PRKG1* gene plays a key role in lipolysis to release fatty acids and glycerol through the hydrolysis of triacyglycerol in adipocytes. We herein considered it as a promising candidate for milk fatty acids. The purpose of this study was to investigate whether *PRKG1* had effects on milk fatty acids.

**Results:**

By direct sequencing the PCR products of pooled DNA, we identified a total of six SNPs, including one in 5′ flanking region, four in 3′ untranslated region (UTR), and one in 3′ flanking region. The single-locus association analysis was carried out, and showed that the six SNPs mainly had significant associations with C6:0, C8:0 and C17:1 (*P* < 0.0001 ~ 0.0035). In addition, we observed a haplotype block formed by g.6903810G > A and g.6904047G > T with Haploview 4.1, and it was strongly associated with C8:0, C10:0, C16:1, C17:1, C20:0 and C16 index (*P* = < 0.0001 ~ 0.0123). The SNP, g.8344262A > T, was predicted to alter the binding site (BS) of transcription factor (TF) GAGA box with Genomatix software, and the subsequent luciferase assay verified that it really changed the transcriptional activity of *PRKG1* gene (*P* = 0.0009).

**Conclusion:**

In conclusion, to our best of knowledge, we are the first who identified the significant effects of *PRKG1* on milk fatty acids in dairy cattle.

**Electronic supplementary material:**

The online version of this article (10.1186/s12863-019-0755-7) contains supplementary material, which is available to authorized users.

## Background

Dairy products are well known for vital nutrients providing high quality protein and energy in human diet [1–3]. The most important economic traits of milk production in dairy cattle include milk yield, fat and protein yield, and fat and protein percentage [4]. Milk fat contains a lot of fatty acids composed of saturated fatty acid (SFA) and unsaturated fatty acid (UFA), and it determines the physiological and sensory properties of the milk [5]. SFA increases the risk of cardiovascular diseases, while UFA decreases the risk [6–11]. For example, C12:0, C14:0 and C16:0 have adverse effects on lower-density lipoprotein cholesterol, that is a risk factor for cardiovascular diseases [10], and substituting n-6 and n-3 polyunsaturated fatty acids for SFAs decreases cardiovascular diseases morbidity and mortality [11]. Fatty acids are regulated by a huge network of genes encoding transcription and translational regulators in living organisms [12], and the heritability of SFA (0.14 ~ 0.33) and UFA (0.08 ~ 0.29) have been reported [13–17].

In dairy cattle, some promising candidate genes and QTL regions for milk fatty acids have been identified in previous Genome-wide association studies (GWASs), such as fatty acid synthase (*FASN*; on BTA19), diacylglycerol O-acyltransferase 1 (*DGAT1*; BTA14), stearoyl-CoA desaturase (*SCD*; on BTA26), 22,833,168 bp to 26,284,743 bp on BTA9 and 1,588,879 bp to 2,764,862 bp on BTA14 [18–23]. Our previous GWAS [23] discovered 83 genome-wide significant single nucleotide polymorphisms (SNPs) and 314 suggestive significant SNPs associated with milk fatty acids in Chinese Holstein cows, in which, one SNP, BTB-01077939 for C10:0 (*P* = 0.000014) and C14 index (*P* = 0.000014), was located within the protein kinase, cGMP-dependent, type І (*PRKG1*) gene, and the other SNP, BTA-11275-no-rs for C12:0 (*P* = 0.000024), was close to the *PRKG1* gene with a distance of 0.85 Mb. In addition, Li et al. reported that a QTL region (1.00 Mbp ~ 27.94 Mbp) on BTA26 is significantly associated with milk fatty acids by performing a joint GWAS based on Chinese and Danish Holstein populations [24]. *PRKG1* gene is located on BTA26 (6,901,760 ~ 8,343,635 bp) spanning about 1442 kb and includes 20 exons. It regulates the lipolysis in adipocytes to release fatty acids and glycerol by the hydrolysis of triacyglycerol. It was reported that *PRKG1* gene was involved in cGMP-PKG signaling pathway to inhibit rat brown adipocyte proliferation [25]. To date, no study has reported the associations of *PRKG1* gene with milk fatty acids in dairy cattle. Hence, the objective of this study was to detect whether the *PRKG1* gene had effects on milk fatty acids. We herein searched potential SNPs in *PRKG1* gene, and examined associations of the identified SNPs with 24 traits in Chinese Holstein cows. Further, we verified the impact of one regulatory SNP on transcriptional activity of *PRKG1* with dual-luciferase assay.

## Results

### SNPs identification

By screening the entire coding region and 5′ and 3′ flanking regions, we identified six SNPs (Table 1) in *PRKG1* gene, including g.8344262A > T in 5′ flanking region, g.6904047G > T, g.6903810G > A, g.6903365C > A and g.6902878 T > G in 3′ untranslated region (UTR), and g.6901713 T > G in 3′ flanking region. The genotypic and allele frequencies of the six SNPs in *PRKG1* gene were shown in Table 1.

**Table 1:** Detailed information of six SNPs identified in *PRKG1* gene

SNP name	Location	Position (UMD 3.1.1)	GenBank no.	Genotype	NO.	Frequency	Allele	Frequency
g.8344262A > T	5′ flanking region	Chr26:8344262	rs109571301	AA	508	0.4824	A	0.6876
				AT	432	0.4103	T	0.3124
				TT	113	0.1073		
g.6904047G > T	3′ UTR	Chr26:6904047	rs478962267	GG	500	0.6729	G	0.8203
				GT	219	0.2948	T	0.1797
				TT	24	0.0323		
g.6903810G > A	3′ UTR	Chr26:6903810	rs444193880	AA	23	0.0219	A	0.1237
				AG	214	0.2036	G	0.8763
				GG	814	0.7745		
g.6903365C > A	3′ UTR	Chr26:6903365	rs42630538	AA	116	0.1950	A	0.2504
				CC	413	0.6941	C	0.7496
				CA	66	0.1109		
g.6902878 T > G	3′ UTR	Chr26:6902878	rs136888798	GG	221	0.3778	G	0.3812
				GT	4	0.0068	T	0.6188
				TT	360	0.6154		
g.6901713 T > G	3′ flanking region	Chr26:6901713	rs381717383	GG	30	0.0286	G	0.1778
				GT	313	0.2984	T	0.8222
				TT	706	0.6730		

Note: *UTR* Untranslated region

### Associations between SNPs/haplotype blocks and 24 milk fatty acids

Genetic associations (significant associations with *P* < 0.0001 ~ 0.0035) of six SNPs were detected for 24 milk fatty acids, and the results were shown in Table 2. The g.8344262A > T was significantly associated with C10:0, C18:1cis-9 and total index. The g.6904047G > T was significantly associated with C17:1 and C17 index. The g.6903810G > A was significantly associated with C8:0, C20:0 and total index. The g.6903365C > A was significantly associated with C6:0, C8:0 and C20:0. The g.6902878 T > G was significantly associated with C6:0, C8:0 and C17:1. The g.6901713 T > G was significantly associated with C6:0, C8:0, C10:0, C17:1, C14 index and C17 index. In addition, as shown in Table 3, the significant dominant (a), additive (d) and allele substitution (α) effects of these six SNPs for C6:0, C8:0, C10:0, C12:0, C14:0, C14:1, C16:0, C16:1, C17:1, C18:1cis-9, C18 index, C20:0, C14 index, C16 index, C17 index, SFA, UFA, SFA/UFA and total index were presented in Table 3 (*P* < 0.05).

**Table 2:** Associations of six SNPs in *PRKG1* gene with milk fatty acid traits (LSM ± SE)

SNP	Genotype (No.)	C6:0 (%)	C8:0 (%)	C10:0 (%)	C11:0 (%)	C12:0 (%)	C13:0 (%)	C14:0 (%)	C14:1 (%)	C15:0 (%)	C16:0 (%)	C16:1 (%)	C17:0 (%)
g.8344262A > T	AA(424–459)	0.4901 ± 0.0122	0.9658 ± 0.0107	2.8472 ± 0.0321^Aa^	0.0577 ± 0.0023	2.9861 ± 0.0418	0.0988 ± 0.0028	10.2891 ± 0.0702	0.6447 ± 0.0180	0.9972 ± 0.0122	34.7950 ± 0.1771	1.3324 ± 0.0248^ab^	0.5664 ± 0.0031
	AT(259–393)	0.4735 ± 0.0125	0.9467 ± 0.0109	2.7685 ± 0.0332^Bb^	0.0581 ± 0.0025	2.9584 ± 0.0423	0.0991 ± 0.0030	10.1625 ± 0.0716	0.6598 ± 0.0187	0.9947 ± 0.0127	34.7954 ± 0.1822	1.3529 ± 0.0257^a^	0.5714 ± 0.0032
	TT(96–104)	0.4769 ± 0.0174	0.9366 ± 0.0150	2.8580 ± 0.0438^ABa^	0.0559 ± 0.0035	2.9889 ± 0.0572	0.0965 ± 0.0047	10.3280 ± 0.0951	0.6555 ± 0.0271	0.9880 ± 0.0188	34.9466 ± 0.2566	1.2643 ± 0.0356^b^	0.5709 ± 0.0047
	*P*	0.2405	0.0177	0.0012^**^	0.8124	0.6385	0.8615	0.0297	0.6391	0.8733	0.7983	0.0295	0.1829
g.6904047G > T	GG(420–458)	0.4580 ± 0.0133	0.9292 ± 0.0119	2.8354 ± 0.0350	0.0580 ± 0.0026	3.0006 ± 0.0449	0.0988 ± 0.0032	10.2605 ± 0.0777^a^	0.6579 ± 0.0199	0.9950 ± 0.0133	34.8344 ± 0.1923	1.2995 ± 0.0272^a^	0.5621 ± 0.0034
	GT(179–196)	0.4682 ± 0.0155	0.9361 ± 0.0131	2.8250 ± 0.0394	0.0584 ± 0.0030	2.9910 ± 0.0502	0.0987 ± 0.0039	10.0941 ± 0.0865^b^	0.6642 ± 0.0229	1.0011 ± 0.0159	34.9128 ± 0.2225	1.3677 ± 0.0312^b^	0.5678 ± 0.0040
	TT(21–24)	0.5161 ± 0.0316	0.8947 ± 0.0258	2.8551 ± 0.0727	0.0538 ± 0.0064	3.0339 ± 0.0974	0.0947 ± 0.0085	10.1180 ± 0.1720^ab^	0.6469 ± 0.0489	0.9967 ± 0.0346	34.7830 ± 0.4581	1.3050 ± 0.0656^ab^	0.5592 ± 0.0085
	*P*	0.1453	0.2466	0.8812	0.7751	0.8935	0.8846	0.0377	0.9145	0.9096	0.9003	0.0304	0.2151
g.6903810G > A	AA(17–21)	0.5425 ± 0.0347	1.0524 ± 0.0269^A^	2.7307 ± 0.0780	0.0547 ± 0.0070	2.8806 ± 0.1040	0.0922 ± 0.0096	10.3798 ± 0.1808	0.6550 ± 0.0523	0.9345 ± 0.0366	34.3377 ± 0.4963	1.2467 ± 0.0677	0.5534 ± 0.0091
	AG(179–191)	0.4720 ± 0.0146	0.9352 ± 0.0127^B^	2.7669 ± 0.0373	0.0580 ± 0.0029	2.9549 ± 0.0484	0.0977 ± 0.0037	10.2389 ± 0.0820	0.6663 ± 0.0225	1.0024 ± 0.0153	34.8727 ± 0.2159	1.3247 ± 0.0301	0.5686 ± 0.0039
	GG(681–741)	0.4849 ± 0.0114	0.9418 ± 0.0102^B^	2.8293 ± 0.0309	0.0592 ± 0.0022	3.0072 ± 0.0393	0.1001 ± 0.0026	10.2294 ± 0.0664	0.6561 ± 0.0169	0.9969 ± 0.0114	34.7215 ± 0.1659	1.3250 ± 0.0233	0.5674 ± 0.0029
	*P*	0.1040	<.0001^**^	0.0464	0.7411	0.1944	0.6019	0.6868	0.8697	0.1831	0.4741	0.4903	0.2478
g.6903365C > A	AA(93–107)	0.4439 ± 0.0206^Aa^	0.9343 ± 0.0169^A^	2.8432 ± 0.0489	0.0586 ± 0.0041	3.0238 ± 0.0635	0.1018 ± 0.0053	10.4947 ± 0.1101	0.6549 ± 0.0305	1.0037 ± 0.0211	34.7232 ± 0.2894	1.2476 ± 0.0410	0.5653 ± 0.0053
	CC(361–381)	0.5229 ± 0.0138^Bb^	0.9924 ± 0.0122^B^	2.8967 ± 0.0367	0.0592 ± 0.0027	3.0341 ± 0.0471	0.0997 ± 0.0033	10.3851 ± 0.0799	0.6661 ± 0.0206	0.9927 ± 0.0140	34.9631 ± 0.2004	1.3330 ± 0.0283	0.5662 ± 0.0035
	CA(49–55)	0.4626 ± 0.0240^ABa^	0.9379 ± 0.0196^A^	2.8406 ± 0.0566	0.0576 ± 0.0048	2.9559 ± 0.0745	0.1039 ± 0.0063	10.1970 ± 0.1278	0.6478 ± 0.0365	0.9746 ± 0.0254	34.2494 ± 0.3514	1.2822 ± 0.0488	0.5598 ± 0.0063
	*P*	<.0001^**^	<.0001^**^	0.2980	0.9415	0.5068	0.7647	0.0958	0.8336	0.6052	0.0875	0.0569	0.5867
g.6902878 T > G	GG(189–202)	0.4323 ± 0.0169^A^	0.9624 ± 0.0144^Aa^	2.8052 ± 0.0423	0.0559 ± 0.0033	2.9744 ± 0.0550	0.0973 ± 0.0042	10.1691 ± 0.0957^a^	0.6547 ± 0.0252	0.9966 ± 0.0172	34.7964 ± 0.2433	1.3831 ± 0.0341	0.5667 ± 0.0044
	GT(2)	0.7892 ± 0.0983^B^	1.3588 ± 0.0820^B^	3.1626 ± 0.2348	0.0560 ± 0.0212	3.0685 ± 0.3132	0.0971 ± 0.0288	11.5294 ± 0.5289^b^	0.5671 ± 0.1627	0.9937 ± 0.1135	34.7693 ± 1.5091	1.1787 ± 0.2088	0.5358 ± 0.0283
	TT(306–334)	0.4942 ± 0.0161^C^	0.9916 ± 0.0137^Ab^	2.8670 ± 0.0409	0.0595 ± 0.0032	3.0380 ± 0.0527	0.1015 ± 0.0040	10.2332 ± 0.0918^a^	0.6368 ± 0.0243	1.0049 ± 0.0166	34.8385 ± 0.2325	1.3627 ± 0.0326	0.5671 ± 0.0042
	*P*	<.0001^**^	<.0001^**^	0.0426	0.4274	0.2875	0.5502	0.0249	0.6194	0.8525	0.9768	0.4788	0.5390
g.6901713 T > G	GG(21–27)	0.5161 ± 0.0328^AB^	1.0577 ± 0.0246^A^	2.8817 ± 0.0710^AB^	0.0539 ± 0.0064	2.8762 ± 0.0928	0.1008 ± 0.0083	10.0821 ± 0.1607^ab^	0.5555 ± 0.0474	0.9906 ± 0.0333	34.1191 ± 0.4451	1.3422 ± 0.0617	0.5699 ± 0.0083
	GT(266–289)	0.4958 ± 0.0132^A^	0.9797 ± 0.0116^B^	2.9305 ± 0.0345^A^	0.0579 ± 0.0026	3.0584 ± 0.0448	0.1000 ± 0.0033	10.3430 ± 0.0756^a^	0.6518 ± 0.0201	0.9971 ± 0.0137	34.8534 ± 0.1956	1.3099 ± 0.0273	0.5649 ± 0.0035
	TT(591–637)	0.4628 ± 0.0116^B^	0.9230 ± 0.0103^C^	2.8342 ± 0.0310^B^	0.0579 ± 0.0023	3.0024 ± 0.0397	0.0982 ± 0.0027	10.1984 ± 0.0673^b^	0.6604 ± 0.0171	0.9930 ± 0.0115	34.9830 ± 0.1682	1.3285 ± 0.0237	0.5711 ± 0.0030
	*P*	0.0029^*^	<.0001^**^	0.0004^**^	0.8083	0.0545	0.8097	0.0181	0.0734	0.9357	0.1082	0.6493	0.1080
SNP	Genotype (No.)	C17:1 (%)	C18:0 (%)	C18:1cis-9 (%)	C18index (%)	C20:0 (%)	C14index (%)	C16index (%)	C17index (%)	SFA (%)	UFA (%)	SFA/UFA (%)	Total index (%)
g.8344262A > T	AA(374–460)	0.1906 ± 0.0025^a^	14.0884 ± 0.0864	18.9343 ± 0.1167^a^	56.9012 ± 0.2735^ab^	0.1685 ± 0.0017	6.0733 ± 0.1384	3.6217 ± 0.0636^AB^	24.8838 ± 0.2156^a^	68.1683 ± 0.1617	30.2498 ± 0.1462	2.2891 ± 0.0212	27.5332 ± 0.1278^A^
	AT(331–393)	0.1957 ± 0.0026^b^	14.0342 ± 0.0907	19.2495 ± 0.1212^b^	57.3243 ± 0.2805^a^	0.1706 ± 0.0017	6.2370 ± 0.1435	3.6885 ± 0.0659^A^	24.9935 ± 0.2245^a^	67.9305 ± 0.1671	30.4415 ± 0.1520	2.2709 ± 0.0221	27.9090 ± 0.1313^B^
	TT(82–104)	0.1889 ± 0.0036^ab^	14.2026 ± 0.1381	18.7636 ± 0.1828^a^	56.3603 ± 0.4109^b^	0.1699 ± 0.0027	6.1182 ± 0.2045	3.4344 ± 0.0913^B^	24.1599 ± 0.3177^b^	68.4212 ± 0.2469	29.9512 ± 0.2243	2.3224 ± 0.0329	27.1715 ± 0.1948^A^
	*P*	0.0218	0.4469	0.0031^*^	0.0323	0.3960	0.3704	0.0101	0.0154	0.0770	0.0628	0.2492	<.0001^**^
g.6904047G > T	GG(382–459)	0.1909 ± 0.0027^Aa^	14.0083 ± 0.0952	19.0595 ± 0.1295	57.3442 ± 0.2994	0.1668 ± 0.0020^a^	6.2722 ± 0.1517	3.6249 ± 0.0698	24.9868 ± 0.2379^A^	68.0210 ± 0.1771	30.3843 ± 0.1611	2.2734 ± 0.0232	27.7337 ± 0.1392
	GT(173–196)	0.1951 ± 0.0031^ABa^	14.0877 ± 0.1147	19.0396 ± 0.1519	56.9780 ± 0.3490	0.1718 ± 0.0023^b^	6.3520 ± 0.1778	3.7536 ± 0.0798	25.1566 ± 0.2723^A^	68.1127 ± 0.2102	30.3983 ± 0.1887	2.2768 ± 0.0275	27.7040 ± 0.1642
	TT(22–24)	0.2129 ± 0.0064^Bb^	14.2950 ± 0.2501	18.9209 ± 0.3330	56.8847 ± 0.7389	0.1642 ± 0.0045^ab^	6.0245 ± 0.3750	3.5526 ± 0.1650	26.8471 ± 0.5501^B^	67.8471 ± 0.4476	30.4643 ± 0.4116	2.2507 ± 0.0598	27.7666 ± 0.3516
	*P*	0.0010^**^	0.4300	0.9118	0.4383	0.0190	0.6321	0.1072	0.0021^*^	0.7890	0.9791	0.9099	0.9710
g.6903810G > A	AA(16–21)	0.1852 ± 0.0068	14.2381 ± 0.2711	19.4313 ± 0.3637	57.8248 ± 0.8116	0.1630 ± 0.0053^AB^	5.8672 ± 0.3861	3.5731 ± 0.1706	25.0782 ± 0.5779	67.6381 ± 0.4921	30.6643 ± 0.4476	2.1938 ± 0.0634	28.8024 ± 0.3790^Aa^
	AG(154–191)	0.1907 ± 0.0030	14.2502 ± 0.1111	18.9316 ± 0.1477	56.9864 ± 0.3386	0.1751 ± 0.0021^A^	6.1871 ± 0.1691	3.6361 ± 0.0768	24.9321 ± 0.2611	68.1679 ± 0.2033	30.2696 ± 0.1845	2.3059 ± 0.0266	27.4558 ± 0.1589^Bb^
	GG(622–743)	0.1907 ± 0.0023	14.0972 ± 0.0800	19.1316 ± 0.1076	57.5775 ± 0.2508	0.1692 ± 0.0016^B^	6.1143 ± 0.1313	3.6545 ± 0.0602	24.8228 ± 0.2058	67.8738 ± 0.1482	30.4623 ± 0.1352	2.2670 ± 0.0196	27.8031 ± 0.1169^ABc^
	*P*	0.6965	0.3037	0.1962	0.1177	0.0027^*^	0.6719	0.8620	0.8107	0.2087	0.4195	0.1027	0.0006^**^
g.6903365C > A	AA(86–107)	0.1840 ± 0.0041	13.8722 ± 0.1550	19.4709 ± 0.2047^a^	57.9027 ± 0.4677	0.1597 ± 0.0031^A^	6.0759 ± 0.2298	3.4734 ± 0.1041	24.7990 ± 0.3588	67.7737 ± 0.2806	30.7073 ± 0.2542^a^	2.2474 ± 0.0368	28.0306 ± 0.2207^a^
	CC(322–383)	0.1898 ± 0.0028	14.0896 ± 0.1003	18.8996 ± 0.1348^b^	57.0679 ± 0.3097	0.1652 ± 0.0020^AB^	6.2036 ± 0.1599	3.6362 ± 0.0727	25.0637 ± 0.2475	68.2830 ± 0.1841	30.0458 ± 0.1674^b^	2.3118 ± 0.0244	27.4913 ± 0.1448^b^
	CA(44–55)	0.1908 ± 0.0049	14.0436 ± 0.1845	19.2701 ± 0.2468^ab^	57.5534 ± 0.5502	0.1736 ± 0.0037^B^	6.2137 ± 0.2664	3.5773 ± 0.1236	25.4468 ± 0.4221	67.8333 ± 0.3333	30.3655 ± 0.3056^ab^	2.2236 ± 0.0451	28.0080 ± 0.2610^ab^
	*P*	0.2709	0.3586	0.0103	0.1432	0.0032^*^	0.8193	0.2198	0.3687	0.0975	0.0221	0.0476	0.0114
g.6902878 T > G	GG(162–202)	0.1990 ± 0.0034^a^	14.1712 ± 0.1255	19.2493 ± 0.1668	57.8214 ± 0.3823	0.1723 ± 0.0025	6.2335 ± 0.1958	3.7811 ± 0.0867	25.7488 ± 0.2926	67.8664 ± 0.2288	30.4505 ± 0.2085	2.2678 ± 0.0303	27.9791 ± 0.1799
	GT(1–2)	0.1396 ± 0.0210^b^	15.1152 ± 0.8492	17.5963 ± 1.1241	55.1627 ± 2.4664	0.2174 ± 0.0205	4.6700 ± 1.1690	3.2808 ± 0.5268	22.3692 ± 1.7890	70.2243 ± 1.4970	27.7265 ± 1.3769	2.6772 ± 0.1992	25.8210 ± 1.1776
	TT(298–334)	0.1937 ± 0.0033^a^	13.9941 ± 0.1205	19.0195 ± 0.1603	57.8556 ± 0.3675	0.1697 ± 0.0024	6.0736 ± 0.1867	3.6985 ± 0.0842	25.3001 ± 0.2887	68.1192 ± 0.2203	30.2583 ± 0.1994	2.2932 ± 0.0291	27.8464 ± 0.1728
	*P*	0.0035^*^	0.1368	0.1045	0.5484	0.0342	0.2492	0.3232	0.0318	0.1353	0.0844	0.0802	0.1353
g.6901713 T > G	GG(23–27)	0.1820 ± 0.0064^AB^	14.2657 ± 0.2465	19.3224 ± 0.3331	57.4973 ± 0.7218	0.1618 ± 0.0046	5.3282 ± 0.3472^a^	3.6417 ± 0.1598	24.7738 ± 0.5396^AB^	67.7758 ± 0.4361	30.6385 ± 0.4050	2.2379 ± 0.0579	28.0440 ± 0.3424
	GT(241–289)	0.1866 ± 0.0027^A^	14.0054 ± 0.0977	18.9266 ± 0.1306	57.1601 ± 0.3031	0.1707 ± 0.0019	6.0394 ± 0.1533^ab^	3.6135 ± 0.0702	24.4844 ± 0.2400^A^	68.2066 ± 0.1805	30.1259 ± 0.1638	2.2896 ± 0.0240	27.4588 ± 0.1422
	TT(524–639)	0.1960 ± 0.0024^B^	14.1040 ± 0.0818	19.1405 ± 0.1105	57.1691 ± 0.2570	0.1715 ± 0.0016	6.2935 ± 0.1335^b^	3.6568 ± 0.0609	25.1479 ± 0.2074^B^	68.0575 ± 0.1524	30.3322 ± 0.1387	2.2695 ± 0.0201	27.7208 ± 0.1193
	*P*	<.0001^**^	0.3793	0.1310	0.8921	0.0925	0.0034^*^	0.7300	0.0019^*^	0.4593	0.2162	0.4936	0.0446

Note: *LSM* least square mean, *SE* standard error. *P* indicates the significances of the association analysis between each SNP and milk fatty acid traits. *P* is the raw value. *: *P* < 0.0083 (the significant association analysis after multiple testing, 0.05/N). ^**^: *P* < 0.0017 (the significant association analysis after multiple testing, 0.01/N). N is the number of SNPs. Different letter (small letters: *P* < 0.05; capital letters: *P* < 0.01) superscripts indicate significant differences among the genotypes. The number in the brackets represents the number of cows for the corresponding genotype

**Table 3:** Additive (a), dominant (d) and substitution (α) effects of six SNPs on milk fatty acid traits

SNP	Genotype	C6:0	C8:0	C10:0	C11:0	C12:0	C13:0	C14:0	C14:1	C15:0	C16:0	C16:1	C17:0
g.8344262A > T	a	0.0066	0.0146^*^	−0.0054	0.0009	− 0.0014	0.0011	− 0.0195	− 0.0054	0.0046	− 0.0758	0.0341^*^	− 0.0022
	d	−0.0100	− 0.0046	− 0.0842^**^	0.0013	− 0.0291	0.0014	− 0.1461^*^	0.0097	0.0021	− 0.0754	0.0546^*^	0.0027
	α	0.0103	0.0163^**^	0.0259	0.0004	0.0094	0.0006	0.0344	−0.009	0.0038	−0.0480	0.0136	− 0.0032
g.6904047G > T	a	−0.0291	0.0172	−0.0098	0.0021	−0.0167	0.0021	0.0712	0.0055	−0.0009	0.0257	−0.0027	0.0015
	d	−0.0189	0.0241	− 0.0203	0.0025	−0.0262	0.0020	−0.0952	0.0119	0.0052	0.1041	0.0655	0.0072
	α	−0.0169	0.0019	0.0032	0.0005	0	0.0008	0.1327^*^	−0.0021	− 0.0042	− 0.0408	−0.0449^*^	− 0.0031
g.6903810G > A	a	0.0288	0.0553^**^	−0.0493	− 0.0022	− 0.0633	−0.0039	0.0752	−0.0005	− 0.0312	− 0.1919	− 0.0391	−0.007
	d	−0.0417^*^	− 0.0620^**^	− 0.0132	0.0010	0.0109	0.0016	−0.0657	0.0108	0.0367	0.3431	0.0388	0.0083
	α	−0.0028	0.0087	−0.0593^*^	− 0.0015	− 0.0551	− 0.0028	0.0255	0.0076	− 0.0035	0.0686	− 0.0099	− 0.0007
g.6903365C > A	a	− 0.0094	− 0.0018	0.0013	0.0005	0.0340	−0.0010	0.1488^*^	0.0036	0.0146	0.2369	−0.0173	0.0027
	d	0.0697^**^	0.0564^**^	0.0548	0.0011	0.0443	−0.0032	0.0392	0.0147	0.0035	0.4768^*^	0.0681^*^	0.0037
	α	−0.0465^**^	− 0.0305^**^	− 0.0264	−0.0001	0.0114	0.0006	0.1288	−0.0039	0.0128	−0.0068	−0.0514	0.0009
g.6902878 T > G	a	−0.0309^**^	−0.0146^**^	− 0.0309^*^	−0.0018	− 0.0318	− 0.0021	− 0.0321	0.0089	− 0.0042	− 0.0211	0.0102	−0.0002
	d	0.3259^**^	0.3817^**^	0.3265	−0.0018	0.0623	−0.0023	1.3282^*^	−0.0786	− 0.0071	− 0.0482	− 0.1943	− 0.0311
	α	0.0458	0.0786^**^	0.0492	−0.0022	−0.0168	− 0.0026	0.2943^*^	− 0.0105	− 0.0059	− 0.0333	−0.0372	− 0.0078
g.6901713 T > G	a	0.0267	0.0674^**^	0.0237	−0.0020	− 0.0631	0.0013	−0.0582	− 0.0524^*^	− 0.0012	− 0.4319^*^	0.0069	− 0.0006
	d	0.0063	−0.0106	0.0726	0.0019	0.1192^*^	0.0005	0.2028^*^	0.0439	0.0052	0.3023	−0.0254	− 0.0056
	α	0.0308^**^	0.0606^**^	0.0703^**^	−0.0008	0.0132	0.0016	0.0710	−0.0244	0.0022	−0.2381	− 0.0094	− 0.0042
SNP	Genotype	C17:1	C18:0	C18:1cis-9	C18 index	C20:0	C14 index	C16 index	C17 index	SFA	UFA	SFA/UFA	Total index
g.8344262A > T	a	0.0009	−0.0571	0.0853	0.2704	− 0.0007	− 0.0224	0.0937^*^	0.3620^*^	− 0.1265	0.1493	−0.0167	0.1808
	d	0.0059^**^	−0.1113	0.4005^**^	0.6935^**^	0.0014	0.1412	0.1605^**^	0.4716^*^	−0.3642^*^	0.3410^*^	−0.0348	0.5566^**^
	α	−0.0013	−0.0154	− 0.0633	0.5280^*^	− 0.0012	−0.0748	0.0333	0.1864	0.0089	0.0224	−0.0037	−0.0262
g.6904047G > T	a	−0.0110^**^	−0.1433	0.0693	0.2298	0.0013	0.1239	0.0362	−0.9301^**^	0.0869	−0.0400	0.0114	−0.0164
	d	−0.0068	−0.0639	0.0494	−0.1365	0.0063^*^	0.2037	0.1649	−0.7603^*^	0.1787	−0.0260	0.0147	−0.0462
	α	−0.0067^**^	−0.1023	0.0378	0.1424	−0.0026	−0.0076	− 0.0700	−0.4462^*^	− 0.0276	−0.0234	0.0020	0.0132
g.6903810G > A	a	−0.0028	0.0705	0.1498	0.1237	−0.0031	−0.1235	− 0.0408	0.1277	− 0.1179	0.1010	− 0.0366	0.4996^**^
	d	0.0028	0.0826	−0.3499	−0.7147	0.0090^**^	0.1963	0.0223	−0.0184	0.4120	−0.2937	0.0755^*^	−0.8470^**^
	α	−0.0007	0.1328	−0.1151	0.6653	0.0038^*^	0.0250	−0.0239	0.1138	0.1943	−0.1214	0.0204	−0.1407
g.6903365C > A	a	−0.0034	−0.0857	0.1004	0.1746	−0.0069^**^	−0.0689	− 0.0520	−0.3239	− 0.0298	0.1709	0.0119	0.0113
	d	0.0024	0.1317	−0.4709^**^	−0.6601	− 0.0014	0.0588	0.1108	−0.0592	0.4795^*^	−0.4906^*^	0.0763^*^	−0.5279^**^
	α	−0.0046	−0.1524	0.3397^*^	0.5108	−0.0062^**^	−0.0986	− 0.1073	−0.2936	− 0.2736	0.4202^*^	− 0.0269	0.2786
g.6902878 T > G	a	0.0027	0.0885	0.1149	−0.0171	0.0013	0.0799	0.0413	0.2243	−0.1264	0.0961	−0.0127	0.0664
	d	−0.0568^**^	1.0326	−1.5381	−2.6758	0.0463^*^	−1.4836	−0.4590	−3.1553	2.2315	−2.6279	0.3967^*^	−2.0918
	α	−0.0111^*^	0.3384	−0.2603	0.6394	0.0150^*^	−0.2903	−0.0694	− 0.5286	0.4180	− 0.5487	0.0854	− 0.4469
g.6901713 T > G	a	−0.0070^*^	0.0808	0.0910	0.1641	−0.0049^*^	−0.4826^**^	− 0.0075	−0.1871	− 0.1409	0.1532	− 0.0158	0.1616
	d	−0.0023	−0.1794	− 0.3048	−0.1731	0.0041	0.2286	−0.0357	−0.4765	0.2900	−0.3594	0.0359	−0.4236^*^
	α	−0.0085^**^	−0.0339	− 0.1048	0.2749	− 0.0023	−0.3369^**^	− 0.0304	−0.4950^**^	0.0448	−0.0772	0.0072	−0.1098

Note: ^*^: *P* < 0.05. ^**^: *P* < 0.01

**Fig. 1:**
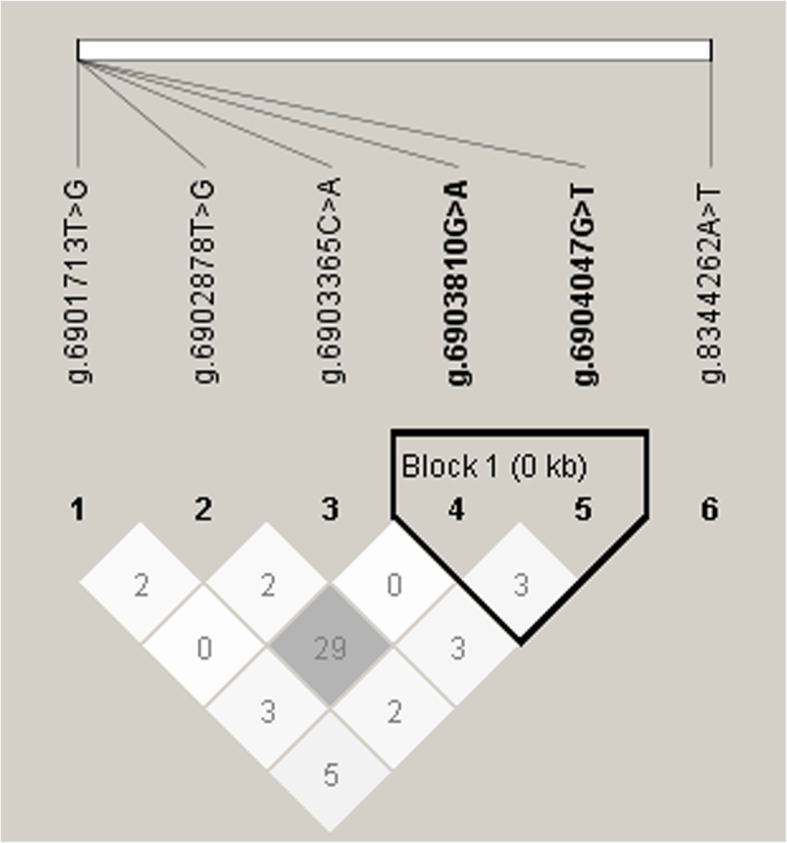
Linkage disequilibrium (LD) among the six SNPs of *PRKG1* gene. r^2^ is the correlation coefficient between the two loci

With the Haploview 4.1, we found a haplotype block (Fig. 1) formed by two SNPs, g.6904047G > T and g.6903810G > A. The haplotype block included haplotypes GG, GT and AG with their frequencies of 67.70, 20.00 and 12.30%, respectively. The haplotype-based association analysis (Table 4) showed that the haplotype block had significant associations with C8:0, C10:0, C16:1, C17:1, C20:0 and C16 index (*P* < 0.0001 ~ 0.0123).

**Table 4:** Associations of haplotype block with milk fatty acid traits (LSM ± SE)

Haplotype combination (No.)	C6:0 (%)	C8:0 (%)	C10:0 (%)	C11:0 (%)	C12:0 (%)	C13:0 (%)	C14:0 (%)	C14:1 (%)	C15:0 (%)	C16:0 (%)	C16:1 (%)	C17:0 (%)
H1H1(425–460)	0.4728 ± 0.0127	0.9386 ± 0.0113^A^	2.8469 ± 0.0338^A^	0.05885 ± 0.0025	3.0339 ± 0.0429	0.1004 ± 0.0030	10.2692 ± 0.0730	0.6462 ± 0.0188	0.9876 ± 0.0128	34.6121 ± 0.1859	1.2967 ± 0.0259^A^	0.5629 ± 0.0033
H1H2(222–241)	0.4565 ± 0.0141	0.9001 ± 0.0123^B^	2.7865 ± 0.0366^AB^	0.05977 ± 0.0028	3.0170 ± 0.0474	0.0988 ± 0.0035	10.2383 ± 0.0808	0.6505 ± 0.0216	1.0003 ± 0.0147	34.9404 ± 0.2085	1.3750 ± 0.0291^B^	0.5701 ± 0.0037
H1H3(131–141)	0.4566 ± 0.0164	0.8898 ± 0.0141^B^	2.7124 ± 0.0412^B^	0.05754 ± 0.0033	2.9655 ± 0.0539	0.0974 ± 0.0042	10.2915 ± 0.0901	0.6679 ± 0.0254	0.9995 ± 0.0173	35.0372 ± 0.2413	1.3139 ± 0.0334^AB^	0.5663 ± 0.0043
*P*	0.2858	<.0001^**^	0.0001^**^	0.8051	0.3027	0.7396	0.8047	0.6499	0.5641	0.0562	0.0064^**^	0.0921
Haplotype combination (No.)	C17:1 (%)	C18:0 (%)	C18:1cis-9 (%)	C18 index (%)	C20:0 (%)	C14 index (%)	C16 index (%)	C17 index (%)	SFA (%)	UFA (%)	SFA/UFA (%)	Total index (%)
H1H1(381–461)	0.1861 ± 0.0026^A^	14.0115 ± 0.0914	19.2062 ± 0.1219	57.4640 ± 0.2836	0.1665 ± 0.0018^A^	6.0911 ± 0.1446	3.5441 ± 0.0670^A^	24.6053 ± 0.2291	67.9358 ± 0.1682	30.4681 ± 0.1529	2.2795 ± 0.0223	27.6825 ± 0.1325
H1H2(204–241)	0.1944 ± 0.0029^B^	14.1742 ± 0.1051	19.1667 ± 0.1413	57.0101 ± 0.3224	0.1739 ± 0.0020^B^	6.2607 ± 0.1663	3.7273 ± 0.0743^B^	24.9757 ± 0.2520	68.1710 ± 0.1925	30.3413 ± 0.1760	2.2958 ± 0.0257	27.5723 ± 0.1512
H1H3(111–141)	0.1894 ± 0.0034^AB^	14.1604 ± 0.1256	19.0181 ± 0.1684	56.9894 ± 0.3797	0.1748 ± 0.0025^B^	6.2861 ± 0.1872	3.5797 ± 0.0862^AB^	24.9042 ± 0.2895	68.1271 ± 0.2277	30.3420 ± 0.2088	2.3025 ± 0.0302	27.3473 ± 0.1793
*P*	0.0037^**^	0.1869	0.4951	0.1820	<.0001^**^	0.3207	0.0123^*^	0.1688	0.3480	0.6593	0.6378	0.1307

Note: *LSM* least square mean, *SE* standard error. *P* indicates the significances of the association analysis between the haplotype block and milk fatty acid traits. *P* is the raw value. ^*^: *P* < 0.05. ^**^: *P* < 0.01. Different letter (small letters: *P* < 0.05; capital letters: *P* < 0.01) superscripts indicate significant differences among the haplotype combinations. The number in the brackets represents the number of cows for the corresponding haplotype combination

**Fig. 2:**
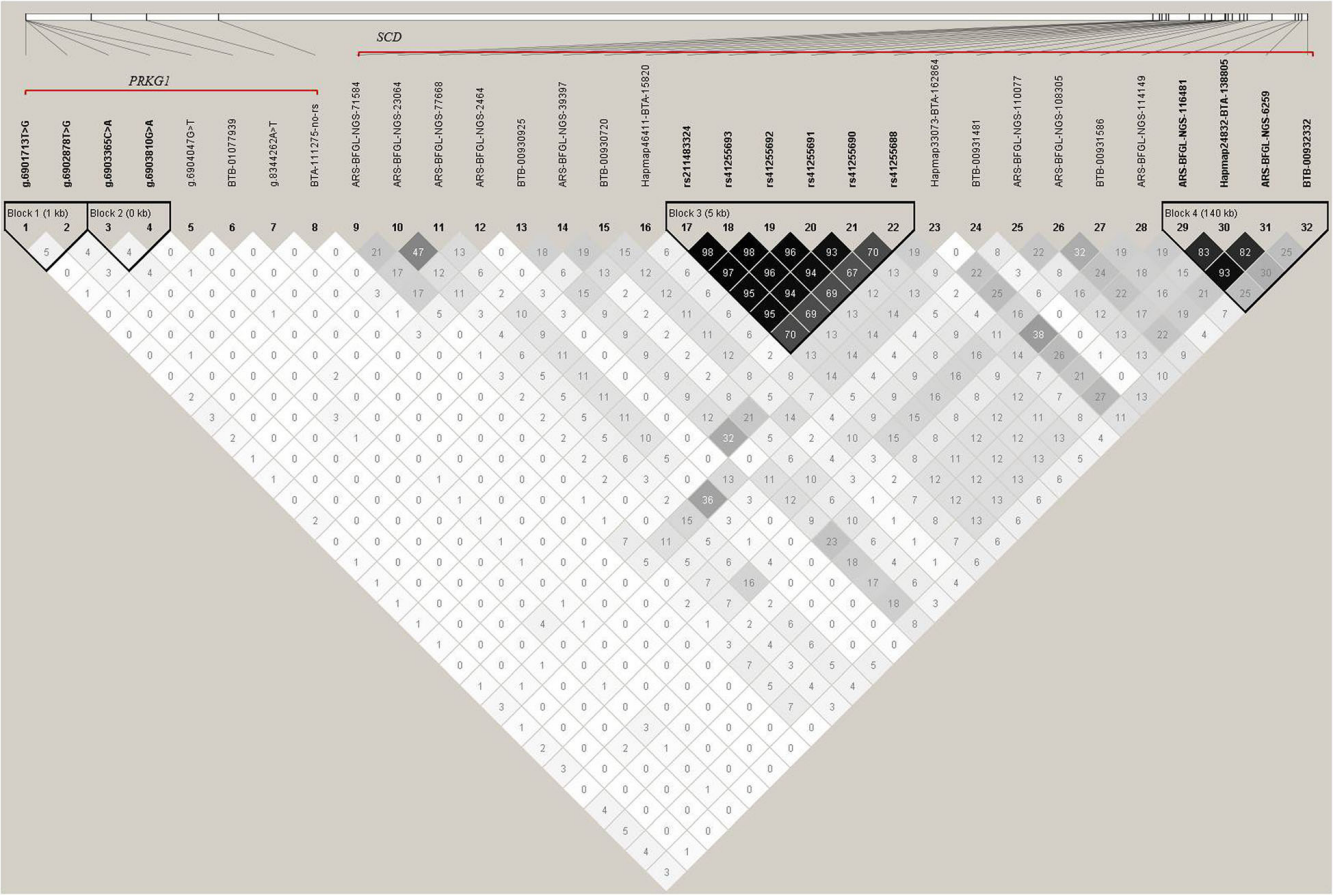
Linkage disequilibrium (LD) among the SNPs of *PRKG1* and *SCD*. r^2^ is the correlation coefficient between the two loci

Further, by estimating the LD among the SNPs of *PRKG1* and *SCD* genes with a distance of 12.79 Mbp, we did not found haplotype block between the two genes (Fig. 2), indicating that the significant effects of *PRKG1* gene on milk fatty acids were not induced by LD with *SCD* gene.

### Change of transcriptional activity caused by g.8344262A > T

We predicted the change of TFBS caused by the SNP in the 5′ flanking region of *PRKG1* gene using Genomatix software, and found that the allele T of g.8344262A > T created a TFBS for GAGA-Box (GAGA).

**Fig. 3:**
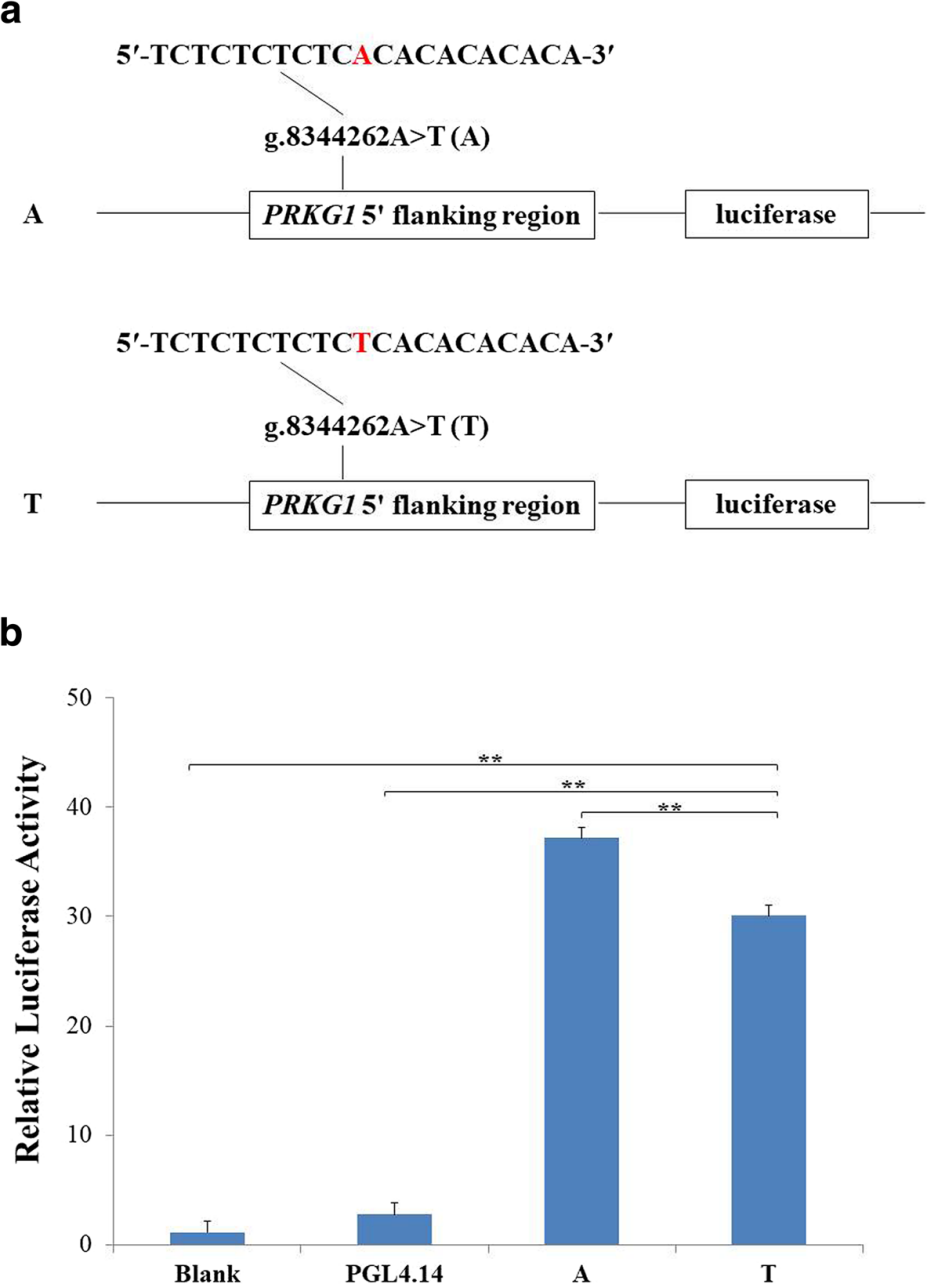
Dual-luciferase assay. **a** Sketches of recombinant plasmids with g.8344262A > T in the 5′ flanking region of *PRKG1* gene. The nucleotides in red highlight referred to the SNP. **b** Luciferase assay analysis of the recombinant plasmids in HEK293 cells. Blank: Blank cells. PGL4.14: Empty vector. A and T: Plasmids of g.8344262A > T. ^**^: *P* < 0.0005

To detect whether g.8344262A > T changed the transcriptional activity of *PRKG1*, we synthesized two constructs with A and T of g.8344262A > T, respectively (Fig. 3a). We measured the luciferases activities of firefly and renilla, and showed the results in Fig. 3b. Luciferase activities of the two constructs were significantly higher than that of the blank control (*P* ≤ 0.0005) and empty vector (PGL4.14; *P* ≤ 0.0006), suggesting that g.8344262A > T might have the transcriptional activity. The allele T of g.8344262A > T had significantly lower luciferase activity than the allele A (*P* = 0.0009), implying that g.8344262A > T could alter the transcriptional activity of *PRKG1* gene.

## Discussion

In our initial GWAS [23], *PRKG1* was considered as one of the promising candidate gene on milk fatty acids in Chinese Holstein. It was also reported that *PRKG1* gene was associated with fatty acid composition in swine [26], and the *PRKG1* knockout mice had lower triglyceride stores in the brown adipose tissue [27]. Here, we first verified that *PRKG1* gene had significant effects on medium-chain saturated fatty acids in dairy cattle, especially C8:0.

*SCD* gene was also a promising candidate gene on BTA26 for milk fatty acids [23], and its effects had been confirmed [28]. In a joint GWAS based on Chinese and Danish Holstein populations, Li et al. also identified a significant QTL region for milk fatty acids (10.00 ~ 27.94 Mbp on BTA26), which included *SCD* and *PRKG1* [24], and the *SCD* was in downstream of *PRKG1* with a distance of 12.79 Mbp. In this study, it was shown that no LD among the SNPs of *PRKG1* and *SCD* was observed, indicating that the effects of *PRKG1* on milk fatty acid traits were independent from *SCD*.

From the KEGG database, we found that *PRKG1* was involved in the cGMP-PKG signaling pathway (ko04022) and regulation of lipolysis in adipocytes (ko04923). In rat, cGMP signaling inhibited brown adipocyte proliferation and thereby promoted brown adipocyte differentiation [25]. The brown adipose tissue from *PRKG1* knockout mice decreased triglyceride stores, suggesting an increase in the ratio of pre-adipocytes to adipocytes and fewer fully differentiated brown adipocytes [27]. In swine, the RNA-Seq analysis identified that the *PRKG1* gene was the differentially-expressed in muscle between high and low groups for fatty acid composition traits [29]. Considering the significant effect of *PRKG1* on milk fatty acid in the present study, we deduced that the gene might have important regulatory function for milk fatty acid metabolism in dairy cattle.

Gene expression is commonly controlled by TFs that are bound to specific sequence elements and the regular regions of the genome [30, 31]. TFBSs are the biding sites (BS) targeted by a DNA binding protein [32], and its disruption is associated with phenotypic diversity [33, 34]. In this study, the allele T of g.8344262A > T was predicted to create a BS for TF GAGA, and it significantly lowered the transcriptional activity of *PRKG1*. GAGA is a drosophila transcription factor involved in many nuclear activities, and can enhance transcription by stabilizing pre-initiation complex and promoting re-initiation [35]. Interactions of GAGA-binding proteins with the GAGA of the *V1bR* promoter activate *V1bR* gene expression, and provide a potential mechanism for physiological regulation of *V1bR* transcription [36]. The significant associations of g.8344262A > T with milk fatty acids, and its impact on transcriptional activity of *PRKG1* gene, suggested that g.8344262A > T might be a potentially causal mutation regulating the *PRKG1* expression by changing the BS for the TF GAGA to affect the formation of milk fatty acids in dairy cattle.

## Conclusions

According to our previous GWAS, we considered *PRKG1* gene as a promising candidate for milk fatty acids in dairy cattle. In the present study, we further confirmed the effects of *PRKG1* on milk fatty acids, and showed that the gene mainly impact on medium-chain saturated fatty acid traits. In addition, we revealed that g.8344262A > T might be a potentially causal mutation altering the transcriptional activity due to the change of a BS for TF GAGA. Our findings might be helpful for the marker-assisted selection in dairy cattle.

## Methods

### Animals and measures of milk fatty acids

We used 1065 Chinese Holstein cows from 44 sire families with an average of 24 daughters per sire for the association analyses in this study. The cows were from 23 dairy farms of Sanyuanlvhe Dairy Farming Center (Beijing, China), a leading dairy company in China, where the standard performance testing for dairy herd improvement (DHI) has been regularly conducted since 1999, and all the cows were fed with the same regular total mixed ration (TMR) composed of concentrated feed and coarse fodder across all subordinate farms. From November to December of 2014, we collected 50 mL milk samples for each cow during 1 ~ 240 days of the first lactation.

Then, we used 2 mL milk samples to measure 16 milk fatty acids (C6:0, C8:0, C10:0, C11:0, C12:0, C13:0, C14:0, C15:0, C16:0, C17:0, C18:0, C20:0, C14:1, C16:1, C17:1 and C18:1cis-9) with the gas chromatography described in the previous GWAS [23] in Beijing Dairy Cattle Center (www.bdcc.com.cn). In addition, we calculated C14 index, C16 index, C17 index, C18 index and total index based on the formulas: $\frac{\mathrm{cis}-9\ \mathrm{unsaturated}}{\mathrm{cis}-9\ \mathrm{unsaturated}+\mathrm{saturated}}\ast 100$ [37], and summarized SFA, UFA, SFA/UFA.

### SNP identification and genotyping

We extracted semen DNAs of 44 Holstein bulls who were sires of the above-mentioned cows using the salt-out procedure, and extracted blood DNAs of the 1065 Chinese Holstein cows with the TIANamp Blood DNA Kit (Tiangen, Beijing, China) according to the manufacturer’s instructions. We measured the quantity and quality of extracted DNA using a NanoDrop™ ND-2000 Spectrophotometer (Thermo Scientific, Hudson, DE, USA) and 2% agarose gel electrophoresis, respectively.

Based on the genomic sequence of the bovine *PRKG1* (Gene ID: 282004), we designed 33 pairs of primers (Additional file 1: Table S1) corresponding the entire exons and 3000 bp of 5′ and 3′ flanking regions using the Primer 3.0 (http://primer3.wi.mit.edu/). The primers were synthesized in Beijing Genomics Institute (Beijing Genomic Institute, Beijing, China). We diluted the genomic DNA of each bull into the concentration of 50 ng/μL, and randomly pooled them into two equal pools (each pool included 22 sire DNAs). The final reaction volume of PCR included 2 μL genomic DNA, 1.25 μL each primer (10 mM), 12.5 μL Premix TaqTM (Takara, Dalian, China) and 8 μL DNase/RNase-Free Deionized Water (Tiangen, Beijing, China).The PCR amplifications for the pooled DNAs were performed, and the procedures were as follows: initial denaturation at 94 °C for 5 min, followed by 35 cycles of 30s at 94 °C, annealing at 60 °C for 30s, extension at 72 °C for 30s, and a final extension at 72 °C for 7 min. Then, we bidirectionally sequenced the PCR products by ABI3730xl DNA analyzer (Applied Biosystems, CA, USA), and aligned them with the bovine reference sequences (UMD 3.1.1) using BLAST (https://blast.ncbi.nlm.nih.gov/Blast.cgi) to search the potential SNPs.

For the SNPs identified in *PRKG1* gene, we used the matrix-assisted laser desorption/ionization time of flight mass spectrometry (MALDI-TOF MS, Sequenom MassARRAY, Bioyong Technologies Inc. HK) to perform the genotyping for the 1065 Chinese Holstein cows.

### Prediction of the transcription factor binding site (TFBS)

We used Genomatix software suite v3.9 (http://www.genomatix.de/cgi-bin/welcome/welcome.pl?s=d1b5c9a9015b02bb3b1a806f9c03293f) to predict the change of TFBS caused by the SNP g.8344262A > T in 5′ flanking region of *PRKG1* gene. In the prediction, we input 30 bp of up/down-stream sequences of g.8344262A > T, namely AGTTTAATATTTATGAAATGTCTCTCTCTC[A]CACACACACACACACACACTCACACGCACA and AGTTTAATATTTATGAAATGTCTCTCTCTC[T]CACACACACACACACACACTCACACGCACA, to research the different transcription factor (TF) bound for allele A and T.

### Recombinant plasmid construction and luciferase assay

In this study, we used the luciferase assay to verify whether the SNP g.8344262A > T changed the transcriptional activity of *PRKG1* gene. We synthesized (Genewiz, Suzhou, China) two fragments (Fig. 3a), A and T of g.8344262A > T, including NheI and HindIII restriction sites at the 5′ to 3′ termini, respectively, and cloned them into the pGL4.14 Luciferase Assay Vector (Promega, Madison, USA). We sequenced these two plasmid constructs to confirm the integrity of the insertions. Then, we purified all the plasmids using the Endo-free Plasmid DNA Mini Kit II (OMEGA, omega bio-tek, Norcross, Georgia, USA).

We cultured Human Embryonic Kidney (HEK)-293 T cells in Dulbecco’s modified Eagle’s medium (DMEM; Gibco, Life Technologies, USA) containing 10% heat-inactivated fetal bovine serum (FBS; Gibco) at 5% CO_2_ and 37 °C. We seeded approximately 2 × 10^5^ cells per well in the 24-well plates, and then used Lipofectamine 2000 (Invitrogen, CA, USA) to transfect the cells according to the manufacturer’s protocol. We transfected 500 ng constructed plasmid DNA along with 10 ng of pRL-TK renilla luciferase reporter vector (Promega) into each well. The experiments were conducted in three replicates for each construct.

We harvested the cells about 48 h after transfection, and measured the activities of firefly and renilla luciferases using a Dual-Luciferase Reporter Assay System (Promega, Madison, USA) on a Modulus microplate multimode reader (Turner Biosystems, CA, USA). We used the average statistics of three replicates as the normalized luciferase data (firefly/renilla).

### Estimation of the linkage disequilibrium (LD)

We estimated the LD among the SNPs identified in *PRKG1* gene in this study using Haploview 4.1 (Broad Institute of MIT and Harvard, Cambridge, MA, USA), and identified the haplotype block. In the process, 95% confidence bounds on D’ were generated and each comparison was called “strong LD”, “inconclusive” or “strong recombination”. If 95% of informative (i.e. non-inconclusive) comparisons were “strong LD”, a block would be created [38]. In addition, we used the r^2^ to represent the correlation coefficient between two loci.

We also performed LD analysis between 8 SNPs in *PRKG1* gene and 24 SNPs in *SCD* gene to detect whether the significant effects of *PRKG1* on milk fatty acids were caused by *SCD*. As the significant SNPs of the two genes identified in two previous studies (Additional file 2: Table S2) [23, 28] were not genotyped in this study, we herein used the database of 1000 Bull Genomes Project [39] to estimate the LD between *PRKG1* and *SCD*. The database of 1000 Bull Genomes Project, including 1575 individuals involved in 48 breeds (Additional file 3: Table S3), was different from our population, while its worldwide correlation implied that the result could be as the indirect support for this study.

### Association analysis

We analyzed the associations between each SNP/haplotype block and 24 milk fatty acids using SAS9.2 software (SAS INSTITUTE Inc., Cary, NC, USA) with the following mixed animal model:

$$ \mathrm{Y}=\mathrm{mu}+\mathrm{herd}+\mathrm{lactation}\ \mathrm{stage}+\mathrm{b}\ast \mathrm{M}+\mathrm{G}+\mathrm{A}+\mathrm{e} $$

For each fatty acid trait, Y is the phenotypic value; mu is the overall mean; herd is the fixed effect of farm; lactation stage is the fixed effect of stage of lactation; M is the fixed effect of calving month; b is the regression coefficient of covariate M; G is the fixed effect corresponding to the genotype or haplotype combination; A is the random polygenic effect that is distributed as N (0, **A** ${\sigma}_a^2$), in which, the numerator relationship matrix (**A**-matrix) was constructed by using Fortran95 code. Pedigree information of the genotyped animals was traced back for three generations. As a result, the total number of animals included in the analysis reaches 3335. In addition, e is the random residual, distributed as N (0, **I** ${\sigma}_e^2$), with identity matrix **I** and residual error variance ${\sigma}_e^2$. The Bonferroni correction for multiple testing was performed based on the number of SNPs. The significant levels of the single SNPs after correction for multiple testing at *P* < 0.05 and *P* < 0.01 were 0.0083 and 0.0017, respectively. In addition, we calculated the additive effect (a), dominant effect (d), and substitution effect (α) using the following formulas [40]: $\alpha =\frac{AA- BB}{2},d= AB-\frac{AA+ BB}{2}, and\ \alpha =\alpha +d\left(q-p\right)$. In which, AA, AB and BB are the least square means of fatty acid traits corresponding to the genotypes, and *p* and *q* are the frequencies of A and B, respectively. Here, the *P* values for significant effects were *P* < 0.05 and *P* < 0.01.

## Additional files

Additional file 1: **Table S1.** PCR primer information of *PRKG1* gene. (XLSX 12 kb)

Additional file 2: **Table S2.** Information of significant SNPs used for estimating the Linkage disequilibrium (LD) between *PRKG1* and *SCD*. (XLSX 10 kb)

Additional file 3: **Table S3.** 48 breeds for 1575 individuals in the database of 1000 Bull Genomes Project. (XLSX 9 kb)

## Data Availability

All relevant data are available within the article and its additional files.

## Abbreviations

a: Additive effect
BS: Binding site
d: Dominant effect
DHI: Dairy herd improvement
GWAS: Genome-wide association study
LD: Linkage disequilibrium
*PRKG1*: Protein kinase, cGMP-dependent, type І
SFA: Saturated fatty acids
SNP: Single nucleotide polymorphism
TF: Transcription factor
TFBS: Transcription factor binding site
UFA: Unsaturated fatty acids
UTR: Untranslated region
α: Substitution effect

## References

[1] Designing foods: animal product options in the marketplace. Washington (DC); 1988. Copyright © 1988 by the National Academy of Sciences. 25032293

[2] Lindsay H. Allen. Animal source foods to improve micronutrient nutrition and human function in developing countries. Proceedings of a conference. June 24-26, 2002. Washington, DC, USA. J Nutr. 2003;133(11 Suppl 2):3875S–4061S. 10.1093/jn/133.11.3875S 14714275

[3] Haug A, Hostmark AT, Harstad OM (2007). Bovine milk in human nutrition--a review. Lipids Health Dis.

[4] Spelman RJ, Coppieters W, Karim L, van Arendonk JA, Bovenhuis H (1996). Quantitative trait loci analysis for five milk production traits on chromosome six in the Dutch Holstein-Friesian population. Genetics.

[5] Illingworth D (1996). Handbook of milkfat fractionation technology and applications : by Kerry E. Kaylegian and Robert C. Lindsay, AOCS press, 1995. $150.00 (xxiv + 662 pages) ISBN 0 935315 57 8. Trends Food Sci Technol.

[6] Briggs Michelle, Petersen Kristina, Kris-Etherton Penny (2017). Saturated Fatty Acids and Cardiovascular Disease: Replacements for Saturated Fat to Reduce Cardiovascular Risk. Healthcare.

[7] Mensink RP, Zock PL, Kester ADM, Katan MB (2003). Effects of dietary fatty acids and carbohydrates on the ratio of serum total to HDL cholesterol and on serum lipids and apolipoproteins: a meta-analysis of 60 controlled trials. Am J Clin Nutr.

[8] Kris-Etherton PM, Pearson TA, Wan Y, Hargrove RL, Moriarty K, Fishell V, Etherton T (1999). High-monounsaturated fatty acid diets lower both plasma cholesterol and triacylglycerol concentrations. Am J Clin Nutr.

[9] Kromhout D, Menotti A, Kesteloot H, Sans S (2002). Prevention of coronary heart disease by diet and lifestyle - evidence from prospective cross-cultural, cohort, and intervention studies. Circulation.

[10] Fernandez ML, West KL (2005). Mechanisms by which dietary fatty acids modulate plasma lipids. J Nutr.

[11] Kris-Etherton PM, Fleming JA (2015). Emerging nutrition science on fatty acids and cardiovascular disease: nutritionists' perspectives. Adv Nutr.

[12] Bionaz M, Loor JJ (2008). Gene networks driving bovine milk fat synthesis during the lactation cycle. BMC Genomics.

[13] Stoop WM, Schennink A, Visker MH, Mullaart E, van Arendonk JA, Bovenhuis H (2009). Genome-wide scan for bovine milk-fat composition. I. Quantitative trait loci for short- and medium-chain fatty acids. J Dairy Sci.

[14] Schennink A, Stoop WM, Visker MH, van der Poel JJ, Bovenhuis H, van Arendonk JA (2009). Short communication: genome-wide scan for bovine milk-fat composition. II. Quantitative trait loci for long-chain fatty acids. J Dairy Sci.

[15] Petrini J, Iung LH, Rodriguez MA, Salvian M, Pertille F, Rovadoscki GA, Cassoli LD, Coutinho LL, Machado PF, Wiggans GR (2016). Genetic parameters for milk fatty acids, milk yield and quality traits of a Holstein cattle population reared under tropical conditions. J Anim Breed Genet = Z Tierzuecht Zuechtungsbiol.

[16] Krag K, Poulsen NA, Larsen MK, Larsen LB, Janss LL, Buitenhuis B (2013). Genetic parameters for milk fatty acids in Danish Holstein cattle based on SNP markers using a Bayesian approach. BMC Genet.

[17] Narayana SG, Schenkel FS, Fleming A, Koeck A, Malchiodi F, Jamrozik J, Johnston J, Sargolzaei M, Miglior F (2017). Genetic analysis of groups of mid-infrared predicted fatty acids in milk. J Dairy Sci.

[18] Olsen HG, Knutsen TM, Kohler A, Svendsen M, Gidskehaug L, Grove H, Nome T, Sodeland M, Sundsaasen KK, Kent MP (2017). Genome-wide association mapping for milk fat composition and fine mapping of a QTL for de novo synthesis of milk fatty acids on bovine chromosome 13. Genet Sel Evol.

[19] Buitenhuis B, Janss LL, Poulsen NA, Larsen LB, Larsen MK, Sorensen P (2014). Genome-wide association and biological pathway analysis for milk-fat composition in Danish Holstein and Danish Jersey cattle. BMC Genomics.

[20] Li X, Buitenhuis AJ, Lund MS, Li C, Sun D, Zhang Q, Poulsen NA, Su G (2015). Joint genome-wide association study for milk fatty acid traits in Chinese and Danish Holstein populations. J Dairy Sci.

[21] Palombo V., Milanesi M., Sgorlon S., Capomaccio S., Mele M., Nicolazzi E., Ajmone-Marsan P., Pilla F., Stefanon B., D'Andrea M. (2018). Genome-wide association study of milk fatty acid composition in Italian Simmental and Italian Holstein cows using single nucleotide polymorphism arrays. Journal of Dairy Science.

[22] Knutsen TM, Olsen HG, Tafintseva V, Svendsen M, Kohler A, Kent MP, Lien S (2018). Unravelling genetic variation underlying de novo-synthesis of bovine milk fatty acids. Sci Rep.

[23] Li Cong, Sun Dongxiao, Zhang Shengli, Wang Sheng, Wu Xiaoping, Zhang Qin, Liu Lin, Li Yanhua, Qiao Lv (2014). Genome Wide Association Study Identifies 20 Novel Promising Genes Associated with Milk Fatty Acid Traits in Chinese Holstein. PLoS ONE.

[24] Li X, Buitenhuis AJ, Lund MS, Li C, Sun D, Zhang Q, Poulsen NA, Su G (2015). Joint genome-wide association study for milk fatty acid traits in Chinese and Danish Holstein populations. J Dairy Sci.

[25] Nisoli E, Clementi E, Tonello C, Sciorati C, Briscini L, Carruba MO (1998). Effects of nitric oxide on proliferation and differentiation of rat brown adipocytes in primary cultures. Br J Pharmacol.

[26] Revilla M, Puig-Oliveras A, Castello A, Crespo-Piazuelo D, Paludo E, Fernandez AI, Ballester M, Folch JM (2017). A global analysis of CNVs in swine using whole genome sequence data and association analysis with fatty acid composition and growth traits. PLoS One.

[27] Amieux PS, McKnight GS (2010). Cyclic nucleotides converge on brown adipose tissue differentiation. Sci Signal.

[28] Li C, Sun D, Zhang S, Liu L, Alim MA, Zhang Q (2016). A post-GWAS confirming the SCD gene associated with milk medium- and long-chain unsaturated fatty acids in Chinese Holstein population. Anim Genet.

[29] Puig-Oliveras Anna, Ramayo-Caldas Yuliaxis, Corominas Jordi, Estellé Jordi, Pérez-Montarelo Dafne, Hudson Nicholas J., Casellas Joaquim, Folch Josep M., Ballester Maria (2014). Differences in Muscle Transcriptome among Pigs Phenotypically Extreme for Fatty Acid Composition. PLoS ONE.

[30] Fuda FS, Karandikar NJ, Chen W (2009). Significant CD5 expression on normal stage 3 hematogones and mature B lymphocytes in bone marrow. Am J Clin Pathol.

[31] Orphanides G, Reinberg D (2002). A unified theory of gene expression. Cell.

[32] Yu CP, Li WH (2017). Predicting transcription factor binding sites and their cognate transcription factors using gene expression data. Methods Mol Biol.

[33] Swinnen G, Goossens A, Pauwels L (2016). Lessons from domestication: targeting Cis-regulatory elements for crop improvement. Trends Plant Sci.

[34] Deplancke B, Alpern D, Gardeux V (2016). The genetics of transcription factor DNA binding variation. Cell.

[35] Vaquero A, Blanch M, Espinas ML, Bernues J (2008). Activation properties of GAGA transcription factor. Biochim Biophys Acta.

[36] Volpi S, Rabadan-Diehl C, Cawley N, Aguilera G (2002). Transcriptional regulation of the pituitary vasopressin V1b receptor involves a GAGA-binding protein. J Biol Chem.

[37] Kelsey JA, Corl BA, Collier RJ, Bauman DE (2003). The effect of breed, parity, and stage of lactation on conjugated linoleic acid (CLA) in milk fat from dairy cows. J Dairy Sci.

[38] Gabriel SB, Schaffner SF, Nguyen H, Moore JM, Roy J, Blumenstiel B, Higgins J, DeFelice M, Lochner A, Faggart M (2002). The structure of haplotype blocks in the human genome. Science.

[39] Daetwyler HD, Capitan A, Pausch H, Stothard P, van Binsbergen R, Brondum RF, Liao X, Djari A, Rodriguez SC, Grohs C (2014). Whole-genome sequencing of 234 bulls facilitates mapping of monogenic and complex traits in cattle. Nat Genet.

[40] Falconer DS, Mackay TFC (1996). Introduction to quantitative genetics.

